# Using Zebrafish for Investigating the Molecular Mechanisms of Drug-Induced Cardiotoxicity

**DOI:** 10.1155/2018/1642684

**Published:** 2018-09-27

**Authors:** Zain Z. Zakaria, Fatiha M. Benslimane, Gheyath K. Nasrallah, Samar Shurbaji, Nadin N. Younes, Fatima Mraiche, Sahar I. Da'as, Huseyin C. Yalcin

**Affiliations:** ^1^Biomedical Research Center, Qatar University, Qatar; ^2^College of Art and Science, Department of Biology, Qatar University, Qatar; ^3^College of Health Sciences, Qatar University, Qatar; ^4^College of Pharmacy, Qatar University, Qatar; ^5^College of Sciences and Engineering, Hamad Bin Khalifa University (HBKU), Qatar

## Abstract

Over the last decade, the zebrafish (*Danio rerio*) has emerged as a model organism for cardiovascular research. Zebrafish have several advantages over mammalian models. For instance, the experimental cost of using zebrafish is comparatively low; the embryos are transparent, develop externally, and have high fecundity making them suitable for large-scale genetic screening. More recently, zebrafish embryos have been used for the screening of a variety of toxic agents, particularly for cardiotoxicity testing. Zebrafish has been shown to exhibit physiological responses that are similar to mammals after exposure to medicinal drugs including xenobiotics, hormones, cancer drugs, and also environmental pollutants, including pesticides and heavy metals. In this review, we provided a summary for recent studies that have used zebrafish to investigate the molecular mechanisms of drug-induced cardiotoxicity. More specifically, we focused on the techniques that were exploited by us and others for cardiovascular toxicity assessment and described several microscopic imaging and analysis protocols that are being used for the estimation of a variety of cardiac hemodynamic parameters.

## 1. Introduction

Zebrafish (*Danio rerio) *is a small tropical fish, native to Southeast Asia and belongs to the minnow family (Cyprinidae) of order Cypriniformes [1]. Over the last 30 years, zebrafish has emerged as a model for studying a variety of human disease development [2]. The zebrafish genome is fully sequenced and its resemblance to human genome is surprisingly high with 87% similarity [3]. It is estimated that 70% of the human genes have orthologue genes in the zebrafish genome [1, 4]. As a result, many genes that are associated with human diseases exist in zebrafish and mutations observed in human patients can be modelled in this animal. The high conservation of zebrafish gene sequence and functions compared to humans further demonstrates how zebrafish can be used to model human diseases including cardiovascular conditions [3]. Besides high gene conservation, the utilization of zebrafish as a model to study cardiovascular development and related disease offers a variety of advantages including optical transparency, rapid cardiovascular development, and a cheaper cost when compared to other* in vivo *models [5].

In addition to investigating gene function and modelling a variety of human disease, zebrafish embryos have been extensively used to study cardiotoxicity [6]. Cardiotoxicity is defined as the toxicity that damages the heart muscle and other cardiac tissues and/or disrupts the electrophysiology of the heart. As a result of cardiotoxicity, the heart may not be able to pump adequate blood throughout the body [7]. If severe, cardiotoxicity may lead to cardiomyopathy in other terms cardiac muscle dysfunction. Cardiotoxicity might occur as a side effect of chemotherapeutical drugs or might develop due to exposure to certain chemicals. Lately, zebrafish embryos were shown to be very useful in toxicology studies, particularly to screen for developmental toxicants [8, 9] and environmental pollutants such as pesticides and heavy metals as well as nanoparticles [10, 11]. Small molecules can be added directly into the fish water within multiwell plates and absorbed via diffusion by the fish. Alternatively, studied agents can be injected directly into the yolk sac [12–15]. Zebrafish is an inexpensive/high throughput experimental model that offers several benefits that include the following: the quantity of the tested agents is reduced; the duration of the experiments is shorter; and technical expertise required for its evaluation is less intensive than that of an equivalent study performed in the traditional animal model, mice [16–18]. Furthermore, chemical libraries can be applied to a large number of embryos to systematically screen for a phenotype of interest.

Drug-induced cardiotoxicity is a leading factor for drug withdrawals from the market and failure of clinical trials. The major factor for this drug attrition is lack of efficacy or drug safety [19–21]. Nowadays, the costs of bringing new drugs to the market are very expensive and are continually increasing. The costs are estimated to be higher than one billion USD per drug [22]. Unexpectedly, this increase in costs does not correlate well with an increased success rate in approving new drugs. Only 10 % of drugs entering phase 1 trial get approved by the United States Food and Drug Administration (FDA) [19, 23]. To reduce the large cost of drug development, there is a need to identify potential adverse drug responses (ADR) as early as possible before entering clinical trials. Academic and pharmaceutical industry researchers are showing an increasing interest in the zebrafish model. Zebrafish is a model that bridges cell culture assays (cost-effective but poor data content) and mammalian models (expensive but high data content) during the preclinical pipeline. The toxicity effects reported from zebrafish-based experiments are considered representative for higher vertebrates including humans. Thus, its use provides a closer scenario to human biology than* in vitro* systems.

In this review, we present the important findings from recent studies that have exploited zebrafish in cardiotoxicity assays. We first explain the basics of heart development in zebrafish and the structure of the cardiovascular system in zebrafish. The following section will discuss the zebrafish cardiotoxicity assays. The last section will involve findings from drug-induced cardiotoxicity and environmental teratogen agent-induced cardiotoxicity. Furthermore, analyzing abnormal cardiac morphologies, and disturbed blood circulation in response to drugs will be highlighted.

## 2. Heart Development and Cardiovascular Structure in Zebrafish

**Figure 1:**
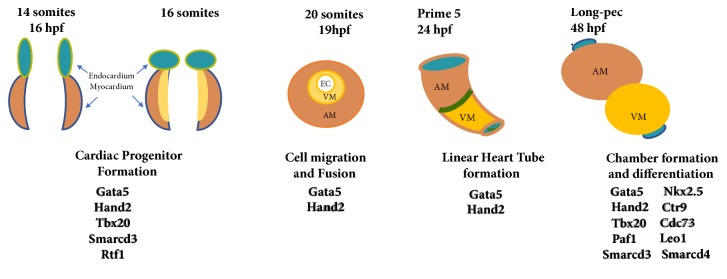
Overview of zebrafish heart development. By 14 hpf, cardiac progenitors have emerged from the anterior lateral plate mesoderm. These cardiac precursors migrate and fuse at the midline to form a cone structure by 19 hpf. After one day of development, a beating linear heart tube has formed to propel circulation through the body. Cardiac chambers are clearly demarcated and looping has completed after two days of development.VM, ventricle myocardium; AM, atrium myocardium; EC, endocardium; (upper panel) stage of development and hour postfertalisation, hpf, and (lower panel) genes that regulate the developmental processes. Gata5, Transcription factor required during cardiovascular development; Hand2, Heart And Neural Crest Derivatives Expressed 2;Tbx20, T-Box Transcription Factor; Smarcd3,4, SWI/SNF Related, Matrix Associated, Actin Dependent Regulator Of Chromatin, Subfamily D, Member 3and 4; CTR1, copper transporter; Cdc37, Cell Division Cycle 37; RTF1, Paf1, LEO1, RNA Polymerase II Complex Component.

In zebrafish, the heart is located anteroventrally to the thoracic cavity between the operculum and the pectoral girdles [27]. The heart is contained in a silver-coloured membranous sac, known as the pericardium. More specifically, within the pericardium, there are four distinct chambers that comprise the heart: the sinus venosus, the atrium, the ventricle, and the outflow tract, called the bulbus arteriosus. However, the fish heart is often referred to as being two-chambered, with one atrium and one ventricle. Heart development starts very early in zebrafish development [28]. At just 5 hours postfertilization (hpf), cardiac progenitors (identified by fate-mapping experiments) are present in the lateral marginal zones of the cleavage stage embryo [29]. Following the gastrulation, cells from the anterior lateral plate mesoderm migrate towards the midline and differentiate into ventricular and atrial cardiomyocytes [29]. Heart tube formation occurs around 24-hpf (Figure 1), at which point the embryo is still translucent, and visualization of ongoing development is possible [30]. At 24-hpf, the heart tube starts to elongate and bends so that the ventricle becomes anterior and the atrium becomes posterior [29]. At this stage, a beating linear heart tube has been formed to propel circulation throughout the body. This movement precedes the looping of the heart, ballooning of the chambers, and formation of the atrioventricular canal. Looping ends around 48-hpf [29]. At 48-hpf, the heart consists of a sinus venosus (the inflow tract that collects blood from cardinal veins and delivers it to the atrium), one atrium (a muscled cavity that receives deoxygenated blood and delivers it to the ventricle), and one ventricle (receives blood from the atrium and delivers it to the body via the aorta). During this time, the bulbus arteriosus (a pear-shaped chamber through which the blood leaves the heart) develops [31, 32]. While the heart is forming, cells are rearranging and beginning to communicate in a way that enables them to begin to pump blood through the heart via contraction. Major morphological events, heart developmental stages, and genes governing heart development in zebrafish are summarized in Figure 1.

## 3. Cardiotoxicity Evaluation in Zebrafish

### 3.1. Cardiac Function Assessment

Zebrafish embryo is a commonly used animal model to investigate the teratogenic effects of the drugs. For this purpose, the cardiovascular function of the animals needs to be evaluated to reveal the influence of exposure on the development of the cardiovascular system as well as on the growth of the whole animal. Simple assays that measure the heart rate currently lack specificity. As such, a more direct evaluation of the cardiac hemodynamic events is critical when zebrafish is used as a robust tool in investigating drug-induced cardiotoxicity. Here we describe microscopy imaging and analysis protocols utilized to calculate a variety of hemodynamic parameters for zebrafish embryos exposed to clinical drugs.

Testing teratogenicity of clinical drugs involves exposing zebrafish embryos to desired concentrations. Due to small size of the embryos, multiple well plates can be used making it possible to test a high number of animals simultaneously [12, 13, 33]. Drug exposure usually begins around 5-hpf, corresponding to late blastula/early gastrula stages and ends at 96-hpf, where most organs are fully developed [34]. In addition to analyzing organ morphologies, blood circulation (hemodynamics) in the exposed animals can also be investigated [35]. Such hemodynamic evaluation enables revealing whether the tested drug affects the whole cardiovascular system, which in turn might affect the development of other systems. For hemodynamic evaluation, the most calculated parameters are heartbeat, cardiac output, fractional area change, fractional shortening, and vascular blood flow velocities [36]. These evaluations can be done by analyzing the embryos under an inverted or a stereo brightfield microscope [37]. In more advanced applications, novel techniques like computational fluid dynamics or particle image velocimetry can also be used for detailed hemodynamic analysis [38, 39].

**Figure 2:**
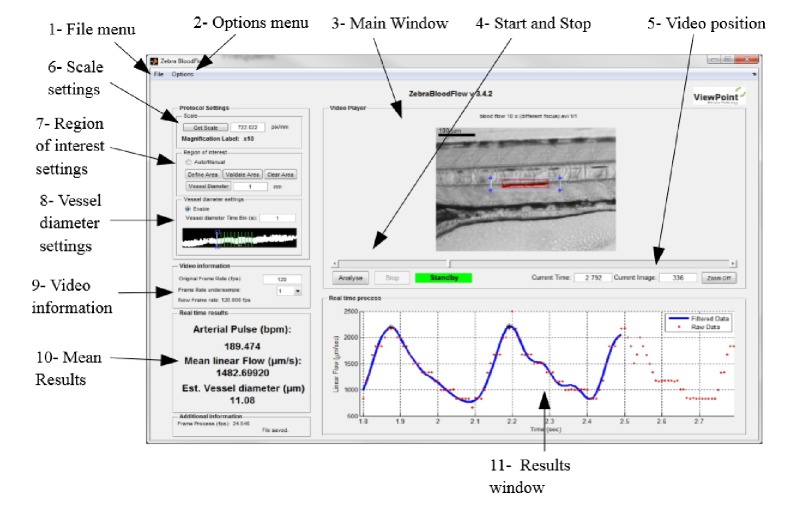
View point's ZebraLab software user interface.

The functional analysis involves quantification of the pumping efficiency of the heart, whereas structural analysis involves precise measurement of heart chamber sizes. These efforts necessitate the utilization of advanced fast imaging techniques like time-lapse microscopy, fluorescent microscopy, and micro-computed tomography. Determination of ventricular wall speeds is important for modelling cardiac muscle conditions such as cardiomyopathy on zebrafish. Levels of wall velocities are about 200–300 *µ*m/sec for 2 to 5 days postfaveolization (dpf) embryos [37]. Therefore, movies should be recorded at high speeds and image analysis is performed on these videos to find ventricular wall speeds and heart rate. Alternatively, a variety of software application can be used to extract the heart rate from beating heart recordings automatically. The first commercially available zebrafish tracking equipment was the ZebraBox/ZebraLab (ViewPoint, France). The system consists of a box with an infrared light source and a camera and it is capable of recording movies with 100 frames per second. The ZebraLab software controls the box and analyzes the swimming activity of the larvae. The infrared light source allows for behavioural monitoring in the dark, as the fish cannot see infrared light [40]. Initially our laboratory used the ZebraBox to analyze larval behaviour, measuring swimming distance, and velocity. However, that initial version of ZebraLab had very limited flexibility with respect to off line data analysis and did not permit analysis of externally recorded movies of zebrafish movement. Current versions of the ZebraBox and the software have been significantly upgraded. The system now has a fully flexible software platform and is capable of analyzing recorded data retrospectively. The aim of the software is to provide information on several cardiac parameters including the heartbeat, blood flow, and vessel diameter variations from a zebrafish high speed video. To evaluate the blood flow, the algorithm determines the correlation between two successive frames (Figure 2). The displacement between the current frame and the best correlated area in the next frame represent the global movement of the blood cells. The heart beat is evaluated by measuring the number of oscillations of the blood flow. To perform the best evaluation, there is an automatic detection of the local minimal value of the first and the last oscillation in the blood flow axis. These values are represented by a black star in the blood flow axis [41]. Determination of cell speed is important for evaluating flow rate for the vessels and estimating the shear stress levels acting on the endothelial cells lining the vessel wall (i.e., shear stress is the frictional force on the endothelial cells from the flowing blood). Levels of red blood cell (RBC) velocities are about 300 *µ*m/sec to 750 *µ*m/sec for 2-5 dpf embryos [42]. Therefore, movies should be recorded at high speeds for further analysis.

**Figure 3:**
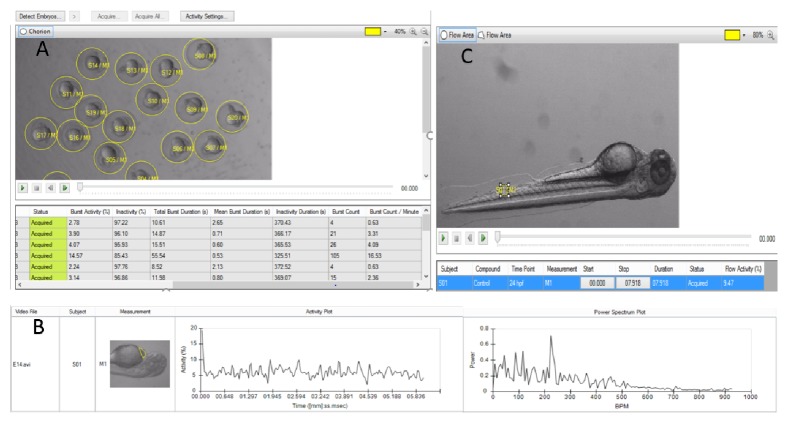
DanioScope system and EthoVision XT9.0 analysis platform. A. DanioScope recognizes the embryos and can analyze videos with multiple animals simultaneously and DanioScope reports back (table A) the following parameters: burst activity (percentage of time the embryo was moving), Inactivity (percentage of time of inactivity); burst duration (total time spent active); inactivity duration (total time spent inactive); burst count (number of times the embryo moved); Burst count/per minute. B. DanioScope measures activity in the heart of each larvae, from this activity the heartbeat in beats per second or per minute is extracted graph B. C. Flow activity can be measured in both blood vessels and the gut.

In 2010, Noldus introduced the DanioVison system (Noldus Information Technology, NL), which consists of the DanioVison chamber for recording the behaviour of larval zebrafish, and the software platform, EthoVision XT9.0, for analyzing the data. EthoVision has a flexible data analysis module, and separately acquired movies can be loaded into the software enabling automated analysis of larvae and adult zebrafish behaviour. Noldus introduced a wide range of tools for all kinds of behavioural tests for zebrafish. The software is used to measure the activity, heartbeat, morphology and flow in embryos and larvae while the video tracking is designed to analyze the movement of adults in several learning, anxiety, or social behaviour paradigms (Figure 3).

Zcardio® (ZeClinics, Spain) has such applications. For example, Zcardio is developed for myocardial fluorescent fish and the software enables for the detection of the following dysfunctions: beat frequency (ventricle and atrium), arrhythmias, QT & QTc interval, fibrillation, ejection fraction, vein blood flow velocity, artery blood flow velocity, and cardiac arrest [43, 44].

### 3.2. Molecular Assessment of Cardiotoxicity

Gene expression profiling may aid in the extrapolation of compound-induced effects for cardiotoxicity investigation, because of the conservation of the molecular pathways and mechanisms between zebrafish and human. In addition to its utility in screening, zebrafish chemical genetics can also help analyze the target and mechanism of action of a test compound through chemical rescue experiments, knockdowns, and phenotype comparisons. Zebrafish are uniquely qualified for use in large-scale screening and the availability of numerous genetic tools facilitates the detailed study of candidate drug effects* in vivo* [32, 45], prior to preclinical testing in mammalian models. The zebrafish chemical genetic screen is a time and cost-effective method for direct* in vivo *drug discovery and serves as an advanced system in drug development [46]. Zebrafish gene expression can be analyzed using tools such as quantitative real-time polymerase chain reaction (qRT-PCR) [47] and in situ hybridization [48]. RNA extraction, followed by RNA microarray analysis is available for gene expression profiling [49]. Western blot and immunohistochemistry can determine the protein expression profiles. Examples of genes or markers (Cardiotoxic genes/proteins) that are screened in cardiotoxicity studies include* SLC28A3, RARG, and UGT1A6 *[50]*. Genetic variants in other genes include the following: ABCC1,2,5 CBR3, CAT, RAC2, HAS3, NCF4, GSTP1, SULT2B1, ABCB1,4, POR, HFE, HAS3, SCL22A17 and NOS3* [51, 52]

## 4. Zebrafish Cardiotoxicity Studies

The zebrafish embryo has been used to assess the impact of exposure of environmental pollutants including Dioxins, PAHs, PBDEs, AChEIs, as well as nanoparticles [53] alcohols [54, 55], and recreational drugs such as Cocaine [56], or Cigarette smoke [57] on the cardiovascular system. Soanes et al. studied the effect of different products of cigarettes on 24 to 48-hpf zebrafish embryos [57]. It was found that zebrafish heartbeat was reduced by 50% at 48-hpf after being exposed to cigarettes [57]. Moreover, continual alcohol exposure (until 2-3 dpf) has been shown to alter the heart of zebrafish functionally and morphologically [58].

Zebrafish was also utilized as a model to assess the cardiotoxicity of several clinical drugs. For instant, cardiotoxicity evaluation of anti-cancer drugs is frequently performed in zebrafish [59]. Cancer patients usually suffer the complications of chemotherapy due to its cardiotoxic effect [55]. Cardiotoxicity has been linked to the morbidity and mortality of cancer patients [60–62], that being said, it can be minimized by accurately identifying the high-risk patients. Identification of patients at high risk can be accomplished through the discovery of new biomarkers. Existing screening methods do not give sufficient results to aid in disease prognosis. In the process of treating cancer with chemotherapy, the cardiological conditions must be considered [63]; this is absent in the current guidelines for cancer treatment. Mostly, chemotherapeutic agents have significant side effects on cardiovascular system which can be prevented by identifying new compounds such as antioxidants and endothelial- or cardiomyocyte-protective agents [60]. Below we explain current findings for the cardiotoxicities of tested agents on zebrafish [64].

### 4.1. Clinical Drug-Induced Cardiotoxicity

One of the widely used anticancer drugs belongs to the Anthracyclines (ANTs) class; Daunorubicin, Pirarubicin, Doxorubicin (DOX), Epirubicin, and DOX-liposome. Han et al. exposed zebrafish embryos to a variety of ANTs that resulted in incomplete looping of the heart tube, pericardial edema and bradycardia in dose-dependent manner [25]. The greatest defect was produced by DOX, whereas Daunorubicin produced the minimum toxicity. ANTs have been shown to downregulate the genes and protein expression related to cardiac development. The ANTs' cardiotoxic effect in the zebrafish model were similar to that reported in other mammalian models [25, 55].

Zhu, et al. showed that zebrafish exhibited pericardial edema and circulatory disturbance in response to cardiotoxic drugs [26]. Seven known human cardiotoxic drugs were tested in the zebrafish model. These included aspirin, clomipramine hydrochloride, cyclophosphamide, nimodipine, quinidine, terfenadine, and verapamil hydrochloride [65]. Aspirin is known to known to elevate the heart rate, and other drugs are known to cause bradycardia. Drugs were administered via soaking or yolk sac microinjection. After 4 and 24 h post-drug treatment, the cardiotoxicity was assessed based on six cardiac parameters: heart rate, heart rhythm, pericardial edema, circulation, hemorrhage, and thrombosis. Evaluation of the effect of human drugs on zebrafish heart led to the conclusion that the effect of these drugs on human and zebrafish are comparable which validated the zebrafish as an excellent model for studying the drug cardiotoxicity [65].

Furthermore, Louis J. D'Amico et al. described methods that are used on the zebrafish for evaluating drug-induced cardiotoxicity [60, 66]. Several drugs from different categories (vancer drugs, antiarrhythmic, anticonvulsant, and beta-blockers) are known to affect the heart function in patients were tested. These drugs included 5-fluorouracil and mitoxantrone, as well as above metnioned drugs; DOX, Cyclophosphamide terfenadine. Drug concentrations ranging from 0.01 *µ*M to 1000 *µ*M were administered to 2 dpf zebrafish embryos for 24 hr. The cardiotoxicity was assessed by measuring the heart rate, heart rhythm, circulation and morphological changes. This study showed similar results as previous [25, 65]; DOX and Cyclophosphamide caused bradycardia while Terfenadine and Clomipramine, which are known to cause a prolongation in QT interval in human [67], induced pericardial edema and hemorrhage in zebrafish. Antibiotics and antiviral drugs such as Gentamicin, Amantadine and Tetracycline rarely induce cardiotoxic effect in humans. These are usually used as a negative control in zebrafish cardiotoxicity studies [68], and as expected they did not induce any cardiac effect in zebrafish [66]. The results were conclusive in employing the zebrafish model for studying drug-induced cardiotoxicity [69, 70].  Table 1 summarizes some findings from this study [66].

Additionally, Cheng et al. assessed the cardiotoxicity of some kinase inhibitors that are used clinically in cancer treatment in zebrafish. The kinase inhibitor, Sorafenib, has been associated with significant declines in ejection fraction in 13% of treated patients [71]. Zebrafish Sorafenib exposure was compared to two kinase inhibitors, Sunitinib that is known to possess cardiotoxic effect and Gefitinib that has no cardiotoxic effect. Zebrafish treatment with Sorafenib and Sunitinib at 5 mol/L induced cardiotoxicity evident by cardiac impairment that was presented with pericardial edema and reduced contractile function. Gefitinib did not result in cardiotoxicity

Moreover, Fang et al. assessed the cardiotoxicity of three addictive drugs, Methamphetamine, Ketamine, and Methadone, in zebrafish embryos [72]. Exposure to 1000 mg/L of Methamphetamine for 12 hours resulted in cardiotoxicity evident with pericardial edema, cardiac looping defects and decreased heart rate in zebrafish. Treatment with the same dose of Ketamine resulted in decreased heart rate. Interestingly, Methadone was lethal at 500 mg/L, while treatment with 100 mg/L resulted in severe circulation abnormalities and pericardial edema. According to these results, Methadone is the most cardiotoxic agents among the three. Based on this data, it was suggestive to advise the clinicians to periodically check the cardiac function of patients treated with these drugs [72].

**Figure 4:**
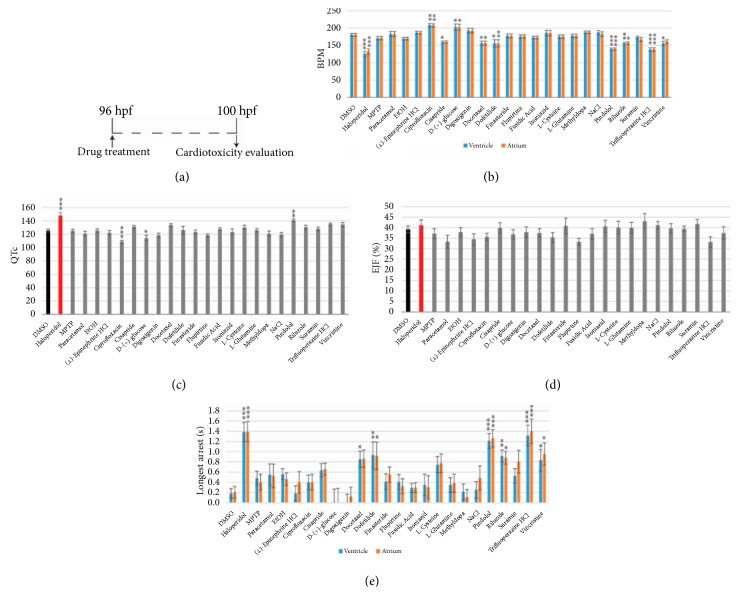
Cardiotoxicity evaluation results adapted from Cornet et al. [24]. (**a**) Scheme of the experimental process; (**b**) bar charts representing the heart beat frequency in beats per minute (bpm); (**c**) QT corrected interval (QTc); (**d**) ejection fraction (EJF); (**e**) and longest cardiac arrest of 100 h old zebrafish larvae.

A study was performed by Cornet et al. to test the toxicity of 24 drugs on zebrafish embryos [24]. Embryos treated with 10 *µ*M haloperidol, known to be cardiotoxic in zebrafish and humans [73], for 4 hrs, were considered as positive controls. Videos were taken under a microscope and ZeCardio® software (ZeClinics) was used to extract different cardiac parameters; heart rate, cardiac arrest, QTc prolongation, and ejection fraction (EJF). Eight compounds out of the twenty four (i.e., haloperidol, cisapride, docetaxel, dofetilide, pindolol, riluzole, trifluoperazine HCL, and vincristine), showed a decrease in the heart rate (bradycardia) and promoted cardiac arrests. In contrast, zebrafish embryos treated with ciprofloxacin and d-glucose showed increased heart rates but no change in the cardiac arrest. Prolongation in the QTc interval was observed in larvae treated with haloperidol and pindolol. However, a shorter QTc interval was observed in the embryos treated with ciprofloxacin and d-glucose.  Figure 4 represents the findings from the study.

**Figure 5:**
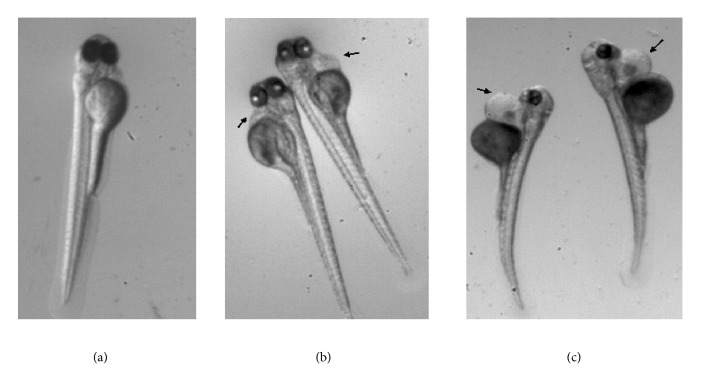
Magnification X= .63. Typical phenotype of a zebrafish embryo incubated from 24-hpf to 96-hpf in** (a)** embryo medium as a negative control, in** (b)** 10 *µ*m diethyl-aminobenzaldehyde (DEAB), and in** (c)** 100 *µ*m DEAB. Note the deformed embryos in DEAB: short size, scoliosis, yolk, and heart edema (black arrows).

Finally, diethyl-aminobenzaldehyde (DEAB) is a drug that we use as a positive control in the toxicological studies in our zebrafish lab at Qatar University Biomedical Research Center. Embryos were incubated from 24-hpf to 96-hpf with 10 *μ*M and 100 *μ*M of DEAB.  Figure 5, shows some cardiological and teratogenic effects after exposure to DEAB.

### 4.2. Cardiotoxic Nanoparticles

Nanotechnology is a multi-trillion-dollar business sector that has an increasing impact on the industrial revolution. Nanoparticles have great potential to be used as drug carriers against human diseases. Zebrafish has been proposed as one of the most successful model and notable advancement in nanotoxicological studies [74]. For instance, at high concentration, gold and silver nanoparticles were shown to change the cardiac morphology in zebrafish [55]. One study has assessed the cardiotoxicity effect of silica nanoparticles using the zebrafish model [75]. Silica nanoparticles have gained great interest for its extensive applications including medical diagnostics, drug delivery and gene therapy [75]. Zebrafish embryos were exposed to silica nanoparticles for continuing exposure period (24 - 96 hpf) to assess the toxicity of silica nanoparticles (embryonic mortality and malformation, cellular death assay). Heart rate was measured at 24 and 48-hpf; significant bradycardia was reported with embryos treated with 100 and 200 *μ*g/mL concentrations compared to lower concentrations of silica nanoparticles and the negative control. In addition to the heart rate measurement, the expression of cardiovascular-related proteins by western blot analysis was used as another approach to assess the cardiotoxic effect of silica nanoparticles. Exposure of the embryos to silica nanoparticles did not cause any significant change in vascular endothelial growth factor receptor 2 (VEGFR2). In contrast, phosphorylated VEGFR2 and angiogenesis-related ERK1/2 expression were inhibited in a dose-dependent manner. Moreover, phosphorylated ERK1/2 expression was inhibited completely. However, no significant changes were observed in ERK1/2, MEF2C/ NKX2.5, and *β*-actin.

Chitosan is a well-known biopolymer that has a wide range of applications including growth inhibition of wide varieties of bacteria and spoilage microorganisms [76]. Recently, our group has shown that chitosan zinc oxide (ZnO) nanoparticles (90% chitosan: 10% ZnO) has inhibitory effect on sulfur reducing bacteria. In the same study and using zebrafish, we showed that these nanoparticles were generally “Green” [77]. In other words, according to Acute Toxicity Rating Scale by Fish and Wildlife Service, Chitosan zinc oxide nanoparticles were regarded as practically nontoxic with LC50 more than 100 *µ*g/mL [78]. The cardiotoxic effects of two nanoparticles were also tested in our zebrafish lab (unpublished results). In these studies, we used Tg[cmlc:GFP] transgenic AB strain, expressing GFP in their cardiac myocyte, to allow good quality of cardiac imaging for heart function assessment. At 96-hpf, we incubated the embryos for 4 hrs. at 28.5°C with (i) the negative control 0.1% DMSO (ii) the positive control 10 *µ*M haloperidol, (iii) 200 *µ*g/mL ChNP, and (iv) 200 *µ*g/mL CZNC. Embryos were then anesthetized by immersion in 0.7 *µ*M Tricaine methanesulfonate and positioned under the microscope to record the videos. We measured the time length of ventricle beat, atrial and ventricular fibrillation, cardiac arrest, ejection fraction [(Ef %) = ((DD-SD)/DD) x100]; DD is the ventricle diastolic diameter (max dilatation); SD is the ventricle systolic diameter (max contraction). For calculation of the QTc interval (linearly corrected QT interval), the Framingham formula (QTc = QT + 0.154 (1 − RR)) was adjusted for zebrafish as QTc = QT + 0.154 (2.66 − RR). RR = 6.6 ms/measured bpm is applied embryos treated with haloperidol showed significant bradycardia and a significant increase in the cardiac arrest compared to the negative control. In contrast, haloperidol-treated embryos showed no significant effect on the ejection fraction, which is expected as there is no evidence in the literature showing that haloperidol causes any significant difference on the ejection fraction. Moreover, embryos treated with haloperidol showed significant Arrhythmia, which was indicated by increase in the percentage of beats defect on the atrium and ventricle compared to the negative control. On the other hand, embryos treated with ChNP and CZNCs showed no significant changes on the heart beats, ejection fraction, percentage of beats defect and cardiac arrest compared to the negative control.

## 5. Conclusions and Future Directions

Because of the conservation between zebrafish and human cardiogenesis, as well as the other advantages such as cost, rapid development, transparency, simple maintenance/egg collection, and ability for survival without a fully functional cardiovascular system, zebrafish became a common model system for cardiovascular disease. As summarized in the previous section, zebrafish models also play a key role in testing different drug cardiotoxicities. The zebrafish model is well conserved and can serve into drug development as a preclinical requirement that can decipher drug safety concerns in patients. Future studies are necessary to uncover the molecular targets of each tested chemical in zebrafish in relevance to cardiac genes/proteins. In each case, an investigation must be done of the known molecular targets of the compound. In addition, the evaluation of whether the compound affects pathways that are known to cause similar phenotypes upon disruption. Finally, if these approaches are not fruitful, a broader search may be needed for novel targets of each compound that are responsible for their effects during cardiovascular development.

## Figures and Tables

**Table 1:** Comparison of cardiotoxicities for several clinical drugs on human and zebrafish.

**Drug**	**Effect on human**	**Effect on zebrafish**
**Doxorubicin [25] ** **5-fluorouracil **Terfenadine [26] **Lidocaine**	Cardiomyopathy, arrhythmia, negative inotropic effects (affect the muscle contraction), or QT prolongation.	Bradycardia, Acute atrioventricular block (AV block), Slow circulation

**Clomipramine [26]**	QT prolongation	Pericardial edema, hemorrhage, bradycardia, and death at higher concentrations

**Quinidine **	QT prolongation	AV block
**Thioridazine**		

**Metoprolol [26]**	Bradycardia	Pericardial edema
**Mexiletine **	Decreased heart rate	Decreased heart rate
**Phenytoin**		
